# Out-of-Hospital Cardiac Arrest Following the COVID-19 Pandemic

**DOI:** 10.1001/jamanetworkopen.2023.52377

**Published:** 2024-01-23

**Authors:** José Ignacio Ruiz Azpiazu, Patricia Fernández del Valle, Alfredo Echarri Sucunza, Jose Antonio Iglesias Vázquez, Carmen del Pozo, Emily Caitlin Lily Knox, Youcef Azeli, Francisco José Sánchez García, Cristian Fernández Barreras, María Carmen Escriche, Pedro Jesús Martín Hernández, Marcos Juanes García, Natividad Ramos García, Sonia Royo Embid, José Antonio Cortés Ramas, Inmaculada Mateo-Rodríguez, Silvia Sola Muñoz, Elena Alcalá-Zamora Marcó, Ana Belén Fornér Canos, Belén Mainar Gómez, Pedro Dacal Pérez, Carmen Camacho Leis, Jose Javier García Cortés, José Manuel Hernández Royano, Xavier Escalada Roig, Antonio Daponte Codina, Fernando Rosell Ortiz

**Affiliations:** 1 Servicio de Servicio de Emergencias 061 de La Rioja, Centro de Investigación Biomédica de La Rioja, Logroño, Spain; 2 Agencia de Servicios Sociales y Dependencia de Andalucía, Sevilla, Spain; 3 Servicio de Urgencias Extrahospitalarias de Navarra, Pamplona, Spain; 4 Fundación Pública Urxencias Sanitarias 061, Galicia, Spain; 5 Emergencias Sanitarias, Castilla y León, Valladolid, Spain; 6 CIBER Epidemiology and Public Health, Granada, Spain; 7 Sistema de Emergencies Mediques, Catalunya, Institut d’ Investigació Sanitaria Pere i Virgili, Tarragona (IISPV), Tarragona, Spain; 8 SUMMA-112 Madrid, Madrid, Spain; 9 Emergentziak-Emergencias, Osakidetza, Spain; 10 SAMU Emergencias Sanitarias, Comunidad Valenciana, Valencia, Spain; 11 Emergencias Sanitarias Extrahospitalarias de Extremadura, ESEX 112, Mérida, Spain; 12 Servicio de Emergencias 061 de La Rioja, Logroño, Spain; 13 SAMUR Protección Civil, Madrid, Spain; 14 061 e Instituto de Ciencias de la Salud, Aragón, Spain; 15 Servicio de Bomberos de Zaragoza, Aragón, Spain; 16 Andalusian School of Public Health, Universidad Nacional a Distancia, CIBER Epidemiology and Public Health, Granada, Spain; 17 Sistema de Emergencies Mediques, Catalunya, Institut d’ Investigació Sanitaria Pere i Virgili, Tarragona, Barcelona, Spain

## Abstract

**Importance:**

Out-of-hospital cardiac arrest (OHCA) health care provision may be a good indicator of the recovery of the health care system involved in OHCA care following the COVID-19 pandemic. There is a lack of data regarding outcomes capable of verifying this recovery.

**Objective:**

To determine whether return to spontaneous circulation, overall survival, and survival with good neurological outcome increased in patients with OHCA since the COVID-19 pandemic was brought under control in 2022 compared with prepandemic and pandemic levels.

**Design, Setting, and Participants:**

This observational cohort study was conducted to examine health care response and survival with good neurological outcome at hospital discharge in patients treated following OHCA. A 3-month period, including the first wave of the pandemic (February 1 to April 30, 2020), was compared with 2 periods before (April 1, 2017, to March 31, 2018) and after (January 1 to December 31, 2022) the pandemic. Data analysis was performed in July 2023. Emergency medical services (EMS) serving a population of more than 28 million inhabitants across 10 Spanish regions participated. Patients with OHCA were included if participating EMS initiated resuscitation or continued resuscitation initiated by a first responder.

**Exposure:**

The pandemic was considered to be under control following the official declaration that infection with SARS-CoV-2 was to be considered another acute respiratory infection.

**Main Outcome and Measures:**

The main outcomes were return of spontaneous circulation, overall survival, and survival at hospital discharge with good neurological outcome, expressed as unimpaired or minimally impaired cerebral performance.

**Results:**

A total of 14 732 patients (mean [SD] age, 64.2 [17.2] years; 10 451 [71.2%] male) were included, with 6372 OHCAs occurring during the prepandemic period, 1409 OHCAs during the pandemic period, and 6951 OHCAs during the postpandemic period. There was a higher incidence of OHCAs with a resuscitation attempt in the postpandemic period compared with the pandemic period (rate ratio, 4.93; 95% CI, 4.66-5.22; *P* < .001), with lower incidence of futile resuscitation for OHCAs (2.1 per 100 000 person-years vs 1.3 per 100 000 person-years; rate ratio, 0.81; 95% CI, 0.71-0.92; *P* < .001). Recovery of spontaneous circulation at hospital admission increased from 20.5% in the pandemic period to 30.5% in the postpandemic period (relative risk [RR], 1.08; 95% CI, 1.06-1.10; *P* < .001). In the same way, overall survival at discharge increased from 7.6% to 11.2% (RR, 1.45; 95% CI, 1.21-1.75; *P* < .001), with 6.6% of patients being discharged with good neurological status (Cerebral Performance Category Scale categories 1-2) in the pandemic period compared with 9.6% of patients in the postpandemic period (RR, 1.07; 95% CI, 1.04-1.10; *P* < .001).

**Conclusions and Relevance:**

In this cohort study, survival with good neurological outcome at hospital discharge following OHCA increased significantly after the COVID-19 pandemic.

## Key Points

**Question:**

Have post–COVID-19 pandemic out-of-hospital cardiac arrest (OHCA) outcomes changed from prepandemic and pandemic levels?

**Findings:**

This cohort study included 14 732 patients with OHCA during prepandemic, pandemic, and postpandemic periods from a Spanish OHCA register. In the postpandemic period, there was a significant increase in recovery of spontaneous circulation at hospital admission and survival at discharge with good neurological outcome.

**Meaning:**

These findings suggest that OHCA care provided by emergency medical systems in Spain is recovering to prepandemic levels.

## Teaser

This cohort study examines whether neurological outcomes among patients with OHCA since the COVID-19 pandemic was brought under control in Spain.

## Introduction

The pandemic brought about by SARS-CoV-2 had a negative collateral effect on health care.^1^ This was a global phenomenon that particularly affected time-dependent conditions, such as stroke, acute myocardial infarction,^2,3,4^ and out-of-hospital cardiac arrest (OHCA). In the case of OHCA, its global incidence is estimated to have increased,^5^ although data are contradictory, depending on several aspects, such as data source and whether OHCA incidence or only resuscitation attempts are reported. Although increased incidence is found in some regions overall in population-based observational studies,^6,7,8,9^ other population-based registries do not reveal substantial changes,^10,11^ with some publications even reporting a decrease in the number of recorded patients with OHCAs with resuscitation attempts from out-of-hospital emergency medical services (EMS).^12^ Agreement does exist regarding the substantial decrease in some of the key actions in the chain of survival, such as bystander defibrillation,^5,12,13^ with even clearer outcomes pertaining to resuscitation attempts. A marked decline has been reported in the percentage of patients with return of spontaneous circulation (ROSC) at hospital arrival and those surviving to hospital discharge.^6,7,8,9,10,11,12,13,14^ This being said, these negative outcomes were found to be independent of local pandemic incidence.^10,12^

The disappearance of such outcomes would provide evidence that normal functioning of health care services has resumed; however, no data are yet available to support this. Survival of patients treated for OHCA, together with key treatment variables associated with survival, may be good indicators of the recovery of the functioning of the health care system involved in the chain of survival. The aim of this study was to identify outcomes pertaining to health care provision for OHCA delivered by EMS as revealed by ROSC status at hospital arrival and survival with good neurological outcome at hospital discharge.

## Methods

This cohort study received approval from the research ethics committees of the health departments of the governments of La Rioja and Navarra. Informed consent was not required because all participating EMS are part of the public health services. They can access the follow-up of the patients they have treated in order to know their own health outcomes, respecting internal legal procedures. The registry has been audited by the Spanish Ministry of Health. This study is reported following the Strengthening the Reporting of Observational Studies in Epidemiology (STROBE) reporting guideline.

We conducted an observational cohort study using the Spanish OHCA register (OHSCAR), a prospective register of consecutive OHCA resuscitation attempts by public EMS in Spain.^15^ Data are collected periodically in noncontinuous time periods. All Spanish EMS are publicly funded and have a physician onboard their ambulances and at their respective dispatch centers.

Inclusion criteria were all consecutive OHCAs in which an EMS team performed resuscitation maneuvers or continued resuscitation or postresuscitation care following cardiopulmonary resuscitation (CPR) attempts by a first responder. OHCAs were excluded if the EMS team suspended resuscitation on-site due to confirmation of futility criteria during resuscitation. A CPR attempt was considered futile when EMS found during resuscitation that CPR was not indicated (eg, terminal disease, unknown or prolonged arrest time prior to EMS arrival, do-not-resuscitate orders). Variable definitions were in line with the Utstein template.^16^

In March 2022, more than 87% of the Spanish population aged older than 5 years was fully vaccinated, and the incidence, hospital admissions, and the severity of COVID-19 cases had been stable for a consistent period of time. This led the Spanish Health Ministry to classify infection by SARS-CoV-2 as just another acute respiratory infection.^17^

To assess the recovery of OHCA health care provision following the COVID-19 pandemic, 3 periods were compared. The pandemic period comprised 3 months, including the 7 weeks corresponding to the first wave of the pandemic (February 1 to April 30, 2020), whereas the prepandemic period was defined as April 1, 2017, to March 31, 2018, and the postpandemic period was defined as January 1 to December 31, 2022. The same EMS and regions participated in all 3 examined periods, with a total population of 28 million inhabitants being covered (eTable in Supplement 1).

### Statistical Analysis

Descriptive statistics are summarized according to the mean (SD), median (IQR), or frequency (percentage), where relevant. Between-group comparisons were made for general patient characteristics, events, and receipt of prehospital and in-hospital care. The Kruskal-Wallis test or analysis of variance was used to make comparisons among continuous variables depending on the distribution of the variable under analysis. Categorical data were compared using the χ^2^ test. Rate ratios in exposed and unexposed groups and 95% CIs were calculated for incidence. Relative risk (RR) was calculated for all remaining variables. All statistical tests were 2-tailed with significance set at *P* < .05. Statistical analyses were performed using R statistical software version 4.3.1 (R Project for Statistical Computing).

The incidence of OHCAs with attempted resuscitation per 100 000 inhabitants per year was adjusted to the duration of data collection periods and to the official censuses of the corresponding regions and years. The proportion of futile resuscitation attempts was examined. Dependent variables were hospital admissions with ROSC, overall survival, and survival with good neurological outcome at discharge, defined as categories 1 and 2 on the Cerebral Performance Category Scale (CPC), where 1 indicates unimpaired or good cerebral performance and 2, moderate disability (disabled but independent).^18^ The 4 subgroups recommended by the Utstein template^16^ were compared.

Segmented regression with a negative binomial distribution to address overfitting and autocorrelation was used to perform 2-way comparisons of the temporal trends in the main outcomes (ROSC on arrival, overall survival, and survival with good neurological outcome) among the 3 examined periods (prepandemic, pandemic, and postpandemic). Time, treatment, and time since treatment coefficients were estimated to determine whether the number of OHCAs changed from 1 examined period to the next. Data analysis was performed in July 2023.

## Results

A total of 15 715 patients were assessed for eligibility. Following the exclusion of 360 patients (5.3%) from the prepandemic period, 147 patients (9.4%) from the pandemic period, and 476 patients (6.4%) from the postpandemic period for being classified as futile resuscitation, a total of 14 732 patients (mean [SD] age, 64.2 [17.2] years; 10 451 [71.2%] male) were entered into the final analysis, with 6372 patients in the prepandemic period, 1409 patients in the pandemic period, and 6951 patients in the postpandemic period. Incidence, general patient characteristics, and administered treatments for all 3 periods are compared in Table 1.

**Table 1.** Descriptive Analysis of General Characteristics of the Study Population and Attention Received

Characteristic	Individuals, No. (%)						Pandemic vs postpandemic		Prepandemic vs postpandemic		Prepandemic vs pandemic	
	Prepandemic		Pandemic		Postpandemic							
	Included	Missing	Included	Missing	Included	Missing	RR (95% CI)	*P* value	RR (95% CI)	*P* value	RR (95% CI)	*P* value
All resuscitations (incidence/100 000 PY)	6372 (23.0)	NA	1409 (20.0)	NA	6951 (24.6)	NA	4.93 (4.6 to 5.22)	<.001	1.07 (1.03 to 1.11)	<.001	0.22 (0.10 to 0.23)	<.001
Sex												
Male	4487 (70.6)	14 (0.2)	1000 (71.0)	1 (0.1)	4964 (71.7)	23 (0.3)	0.99 (0.97 to 1.02)	.63	0.98 (0.94 to 1.01)	.17	0.98 (0.88 to 1.10)	.74
Female	1871 (29.4)	14 (0.2)	408 (29.0)	1 (0.1)	1964 (28.3)	23 (0.3)						
Age, y												
Mean (SD)	64.6 (17.2)	31 (0.5)	64.4 (16.6)	3 (0.2)	63.8 (17.3)	27 (0.4)	0.56 (−0.39 to 1.52)^a^	.25	0.78 (0.19 to 1.37)	.01	0.22 (−0.75 to 1.18)^a^	.66
≤14	103 (1.6)	31 (0.5)	21 (1.5)	3 (0.2)	107 (1.5)	27 (0.4)	1.11 (1.07 to 1.15)	<.001	1.36 (1.27 to 1.46)	<.001	0.93 (0.63 to 1.38)	.73
≥75	2034 (32.1)	31 (0.5)	428 (30.4)	3 (0.2)	2056 (26.7)	27 (0.4)	0.99 (0.97 to 1.01)	.58	0.95 (0.91 to 0.98)	.003	0.93 (0.84 to 1.05)	.23
OHCA at home	3766 (59.1)	0	959 (68.1)	0	4197 (60.4)	0	0.94 (0.93 to 0.96)	<.001	1.03 (0.99 to 1.07)	.13	1.37 (1.24 to 1.53)	<.001
OHCA witnessed	4931 (77.4)	0	1106 (78.5)	0	5482 (78.9)	0	1.00 (0.98 to 1.03)	.76	1.04 (1.00 to 1.09)	.04	1.05 (0.94 to 1.18)	.90
CPR performed before EMS arrival^b^												
Any	2633 (47.9)	0	523 (45.3)	0	3534 (60.3)	0	1.11 (1.08 to 1.13)	<.001	1.28 (1.23 to 1.33)	.01	0.92 (0.82 to 1.02)	.10
Performed by bystander	1770 (32.2)	0	337 (29.2)	0	2443 (41.7)	0	1.09 (1.07 to 1.11)	<.001	1.21 (1.17 to 1.25)	.01	0.89 (0.79 to .99)	.05
Performed by non-EMS personnel	565 (10.3)	0	92 (8.0)	0	549 (9.4)	0	1.03 (0.99 to 1.06)	.11	0.95 (0.89 to 1.01)	.11	0.79 (0.65 to 0.96)	.02
Performed by other public service personnel	298 (5.4)	0	94 (8.1)	0	542 (9.2)	0	1.02 (0.99 to 1.06)	.21	0.30 (0.28 to 0.33)	.01	0.18 (0.15 to 0.22)	.01
AED used before EMS arrival	752 (13.7)	0	110 (9.5)	0	753 (12.8)	0	1.33 (1.11 to 1.61)	<.001	1.04 (0.98 to 1.09)	.18	1.06 (1.03 to 1.09)	<.001
AED with shock												
Any	306 (5.6)	0	43 (3.7)	0	400 (6.8)	0	1.75 (1.30 to 2.33)	<.001	1.12 (1.03 to 1.22)	.01	0.94 (0.90 to 0.98)	<.001
Performed by bystander	131 (2.4)	0	13 (1.1)	0	140 (2.4)	0	1.96 (1.16 to 3.33)	.01	1.01 (0.88 to 1.14)	.99	0.91 (0.86 to 0.95)	<.001
Performed by non-EMS personnel	126 (2.3)	0	19 (1.6)	0	121 (2.1)	0	1.22 (0.80 to 1.85)	.36	0.94 (0.84 to 1.08)	.39	0.95 (0.89 to 1.01)	.12
Performed by other public service personnel	49 (0.9)	0	11 (1.0)	0	129 (2.2)	0	2.13 (1.20 to 3.70)	.01	1.79 (1.39 to 2.27)	<.001	1.01 (0.89 to 1.14)	.85
AED without shock												
Any	446 (8.1)	0	67 (5.8)	0	353 (6.0)	0	1.03 (1.30 to 4.35)	.77	0.85 (0.80 to 0.92)	<.001	0.94 (0.92 to 0.98)	<.001
Performed by bystander	159 (2.9)	0	18 (1.6)	0	90 (1.5)	0	0.99 (0.65 to 1.52)	.95	0.75 (0.68 to 0.83)	<.001	0.92 (0.87 to 0.96)	<.001
Performed by non-EMS personnel	236 (4.3)	0	29 (2.5)	0	117 (2.0)	0	0.83 (0.59 to 1.15)	.25	0.72 (0.66 to 0.78)	<.001	0.93 (0.88 to 0.96)	<.001
Performed by other public service personnel	51 (0.9)	0	20 (1.7)	0	133 (2.3)	0	1.27 (0.84 to 1.92)	.26	1.75 (1.39 to 2.22)	<.001	1.15 (0.99 to 1.33)	.06
Shockable initial rhythm	1463 (23.8)	230 (3.6)	276 (20.6)	68 (4.8)	1552 (22.9)	184	1.15 (0.99 to 1.33)	.06	0.98 (0.94 to 1.55)	.24	0.86 (0.75 to 0.96)	.01
Airway management												
Orotracheal intubation	4569 (71.7)	1770	835 (59.3)	0	4884 (70.3)	0	1.09 (1.07 to 1.12)	<.001	0.96 (0.93 to 1.00)	.06	0.64 (0.58 to 0.70)	<.001
Supraglottic device	312 (4.9)	0	162 (11.5)	0	759 (10.9)	0	0.99 (0.96 to 1.02)	.53	1.40 (1.34 to 1.46)	<.001	2.00 (1.75 to 2.29)	<.001
Call to ambulance arrival time, min^b^												
Median (IQR)	13.0 (8.7 to 19.7)	369 (6.8)	14.1 (9.5 to 21.4)	282 (24.4)	14.6 (9.9 to 22.0)	460 (7.8)	2.85 (0.85 to 4.95)	.01	1.30 (−0.15 to 2.08)^a^	.09	−1.85 (−4.00 to 0.20)^a^	.08
≤8	1044 (20.8)	369 (6.8)	144 (16.5)	282 (24.4)	897 (16.6)	60 (7.8)	0.99 (0.96 to 1.02)	.43	0.85 (0.80 to 0.89)	<.001	0.80 (0.68 to 0.95)	.01
≤15	2984 (59.3)	369 (6.8)	507 (58.1)	282 (24.4)	3044 (56.4)	60 (7.8)	0.97 (0.95 to 0.99)	<.001	0.89 (0.87 to 0.93)	<.001	0.99 (0.88 to 1.13)	.94

Abbreviations: AED, automated external defibrillator; CPR, cardiopulmonary resuscitation; EMS, emergency medical service; OHCA, out-of-hospital cardiac arrest; RR, rate ratio.

^a^ Expressed as odds ratio (95% CI).

^b^ OHCAs attended to by EMS were excluded from this analysis.

**Figure 1.**
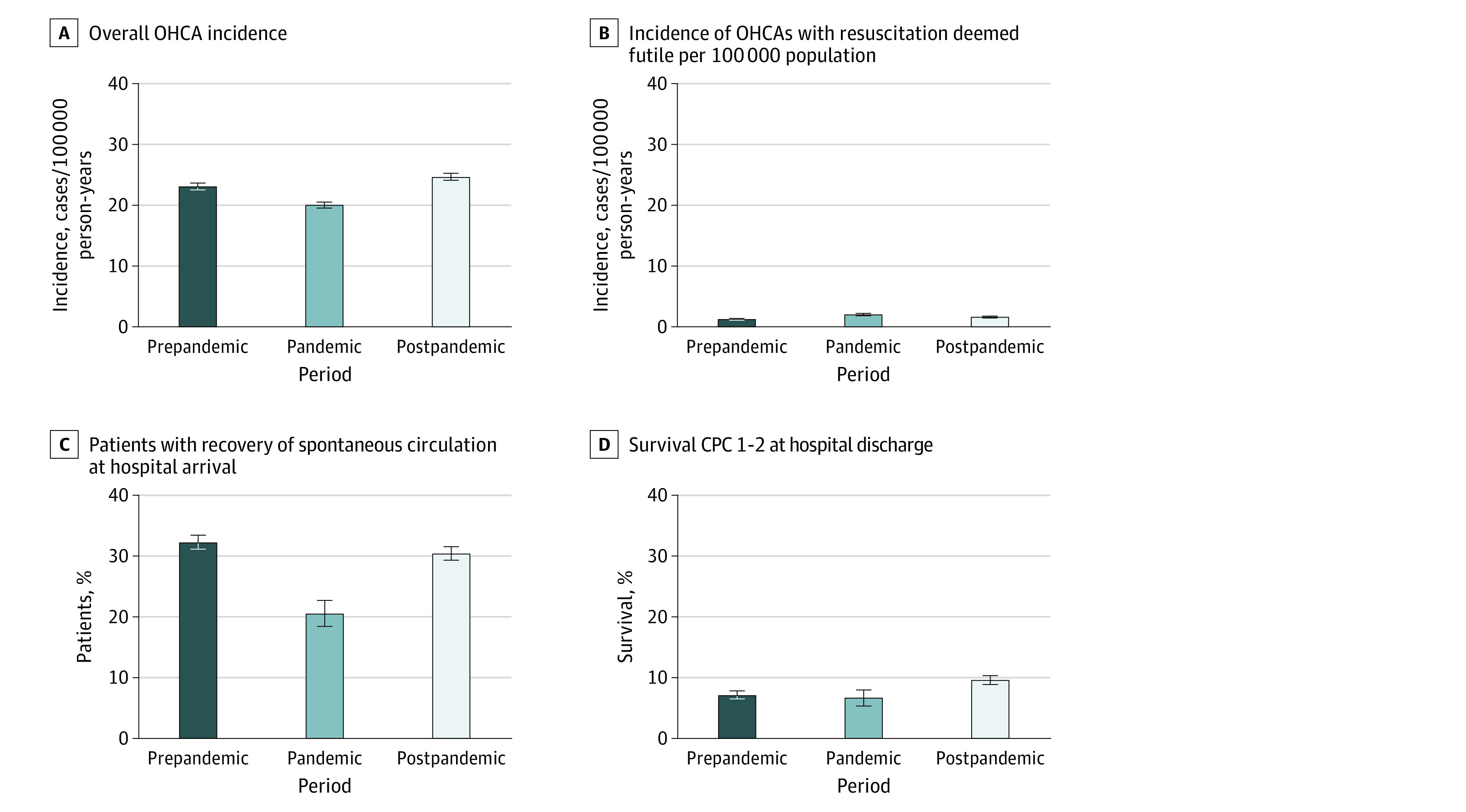
Out-of-Hospital Cardiac Arrest Incidence (OHCA) and Outcomes CPC indicates Cerebral Performance Category. Categories 1 and 2 are considered good neurological outcomes.

Compared with the pandemic period, the postpandemic period had a higher incidence of OHCAs with a resuscitation attempt (20.0 per 100 000 person-years vs 24.6 per 100 000 person-years; RR, 4.93; 95% CI, 4.66-5.22; *P* < .001), whereas the incidence of futile resuscitations was lower (2.1 per 100 000 person-years vs 1.3 per 100 000 person-years; RR, 0.81; 95% CI, 0.71-0.92; *P* < .001) (Figure 1, A and B). The proportion of OHCAs occurring at home decreased from 68.1% to 60.4% (RR, 0.94; 95% CI, 0.93-0.96; *P* < .001), whereas the number of OHCAs with bystander CPR before EMS arrival increased (29.2% vs 41.7%; RR, 1.09; 95% CI, 1.07-1.11; *P* < .001). There was no significant change in OHCAs with a shockable initial rhythm (20.6% vs 22.9%; RR, 1.15; 95% CI, 0.99-1.33; *P* = .06). The use of automated external defibrillator (AED) before EMS arrival increased overall (9.5% vs 12.8%; RR, 1.33; 95% CI, 1.11-1.61; *P* < .001), when applied with shock (3.7% vs 6.8%; RR, 1.75; 95% CI, 1.30-2.33; *P* < .001), and when the AED with shock was applied by a bystander (1.1% vs 2.4%; RR, 1.96; 95% CI, 1.16-3.33; *P* = .01) (Table 1).

The use of endotracheal intubation for airway management before hospital arrival increased significantly, from 59.3% to 70.3% (RR, 1.09; 95% CI, 1.07-1.12; *P* < .001). No significant changes emerged regarding hospital treatment, with the exception of implantable cardioverter-defibrillator implants at hospital discharge, which increased from 4.2% to 11.0% (RR, 1.10; 95% CI, 1.06-1.13; *P* < .001).

**Figure 2.**
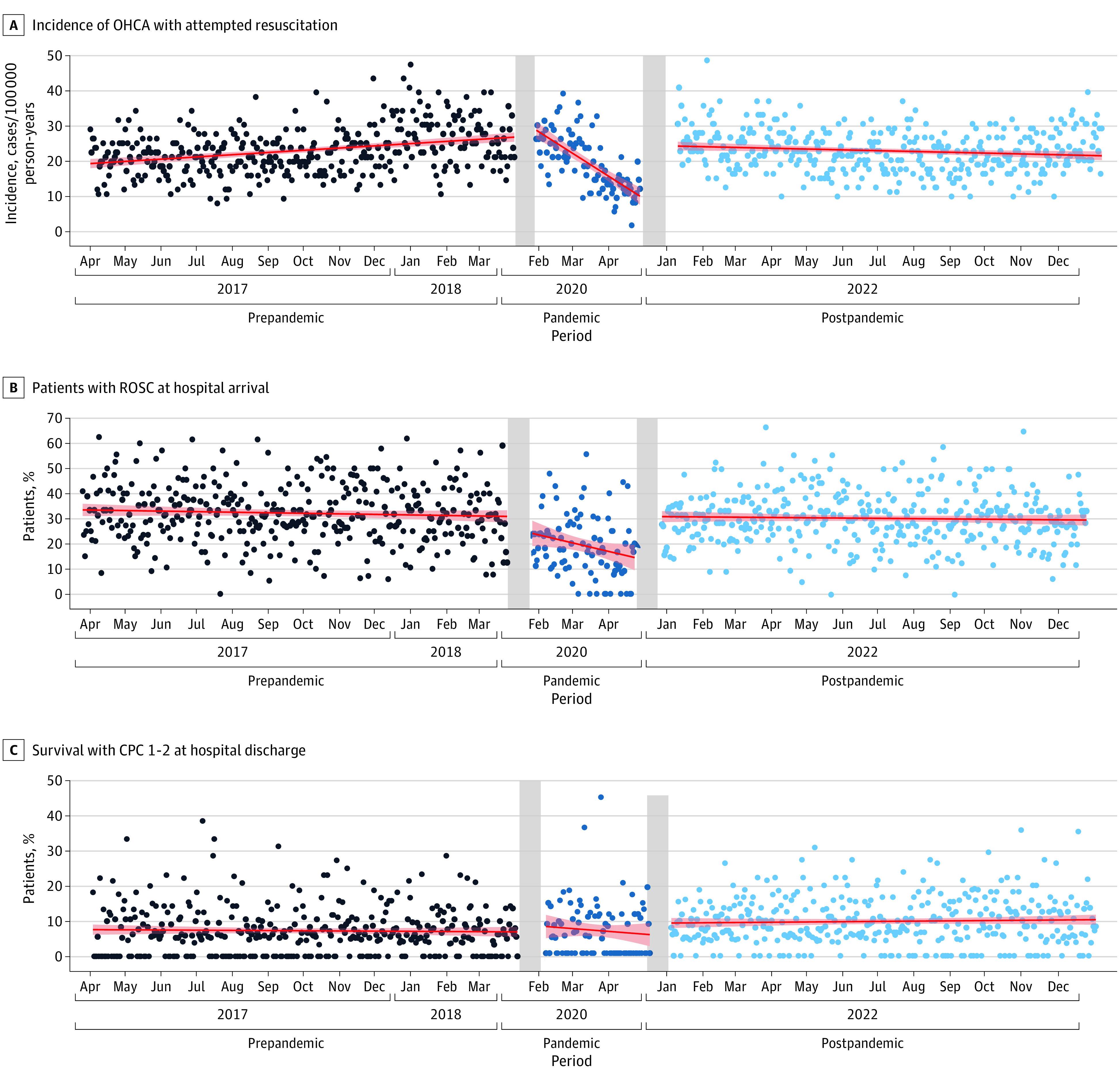
Comparisons of the Temporal Trends in Main Outcomes, Examined via Segmented Regression CPC indicates Cerebral Performance Category (categories 1 and 2 are considered good neurological outcomes); OHCA, out-of-hospital cardiac arrest; ROSC, recovery of spontaneous circulation.

With regard to final outcomes (Table 2), the proportion of patients with ROSC at hospital admission increased from 20.5% to 30.4% (RR, 1.08; 95% CI, 1.06-1.10; *P* < .001). Overall survival at discharge increased from 106 patients (7.6%) in the pandemic period to 781 patients (11.2%) in the postpandemic period (RR, 1.45; 95% CI, 1.21-1.75; *P* < .001), with 670 patients (9.6%) being discharged with good neurological status (ie, CPC category 1-2) in the postpandemic period vs 93 patients (6.6%) in the pandemic period (RR, 1.07; 95% CI, 1.04-1.10; *P* < .001) (Figure 1, C and D, and Figure 2).

**Table 2.** Comparative Analysis of OHCA Care Outcomes in the Included Periods

Outcome	Individuals, No. (%)						Pandemic vs postpandemic		Prepandemic vs postpandemic		Prepandemic vs pandemic	
	Prepandemic		Pandemic		Postpandemic							
	Included	Missing	Included	Missing	Included	Missing	RR (95% CI)	*P* value	RR (95% CI)	*P* value	RR (95% CI)	*P* value
ROSC at hospital	2048 (32.2)	18 (0.3)	287 (20.5)	12 (0.8)	2113 (30.4)	0	1.08 (1.06-1.10)	<.001	0.96 (0.93-0.99)	.03	0.59 (0.53-0.68)	<.001
Shockable initial rhythm,	793 (40.3)	72 (3.5)	122 (44.9)	15 (1.1)	837 (41.3)	85 (4.0)	0.98 (0.95-1.01)	.27	1.02 (0.96-1.09)	.50	1.18 (0.95-1.48)	.14
Ongoing CPR at hospital	184 (2.9)	18 (0.3)	101 (7.2)	12 (0.8)	117 (3.3)	0	0.27 (0.25-0.30)	<.001	1.20 (1.11-1.28)	<.001	2.04 (2.73-2.41)	<.001
Donation in asystole	73 (1.2)	18 (0.3)	7 (0.5)	12 (0.8)	21 (0.6)	0	1.03 (0.91-1.15)	.67	0.69 (0.54-0.88)	<.001	0.48 (0.24-0.98)	.04
In-hospital treatment^a^												
PCI treatment	426 (20.8)	0	83 (28.9)	0	710 (33.6)	0	1.02 (0.99-1.06)	.24	1.35 (1.27-1.43)	<.001	0.29 (0.24-0.36)	<.001
Thrombolysis treatment	25 (1.2)	0	5 (1.7)	0	54 (2.6)	0	1.04 (0.96-1.13)	.32	1.36 (1.16-1.58)	<.001	1.36 (0.61-3.06)	.45
TTM	122 (6.0)	0	23 (8.0)	0	169 (8.0)	0	1.00 (0.95-1.06)	.99	1.16 (1.04-1.28)	.01	1.32 (0.89-1.95)	.17
ICD implant	95 (4.6)	0	12 (4.2)	0	233 (11.0)	0	1.10 (1.06-1.13)	.001	1.44 (1.34-1.56)	<.001	0.91 (0.53-1.57)	.73
Survival at hospital discharge												
Overall	644 (10.1)	0	106 (7.6)	12 (0.9)	781 (11.2)	0	1.45 (1.21-1.75)	<.001	1.06 (1.01-1.14)	.04	0.95 (0.92-0.98)	.01
CPC category												
1-2	455 (7.1)	0	93 (6.6)	12 (0.9)	670 (9.6)	0	1.07 (1.04-1.10)	<.001	1.22 (1.16-1.29)	<.001	0.99 (0.82-1.21)	.99
1	387 (6.1)	0	69 (4.9)	12 (0.9)	587 (8.4)	0	1.09 (1.06-1.12)	<.001	1.19 (1.13-1.25)	<.001	0.83 (0.66-1.04)	.09
2	68 (1.1)	0	24 (1.7)	12 (0.9)	83 (1.2)	0	0.94 (0.84-1.04)	.21	1.07 (0.92-1.24)	.37	1.45 (1.03-2.06)	.04
3	43 (0.7)	0	7 (0.5)	12 (0.9)	67 (1.0)	0	1.10 (1.02-1.18)	.02	1.18 (1.01-1.38)	.02	1.23 (0.42-3.61)	.70
4	39 (0.6)	0	5 (0.4)	12 (0.9)	38 (0.5)	0	1.07 (0.96-1.19)	.24	0.96 (0.76-1.20)	.71	0.63 (0.27-1.44)	.27
Alive, unknown neurological status	107 (1.7)	0	1 (0.1)	12 (0.9)	6 (0.1)	0	1.04 (0.77-1.40)	.82	0.10 (0.05-0.22)	<.001	0.05 (0.01-0.36)	<.001

Abbreviations: AED, automated external defibrillator; CPC, cerebral performance category; CPR, cardiopulmonary resuscitation; EMS, emergency medical service; ICD, implantable cardioverter-defibrillator; PCI, percutaneous coronary intervention; OHCA, out-of-hospital cardiac arrest; ROSC, return of spontaneous circulation; RR, relative risk; TTM, targeted temperature management.

^a^ Refers to patients with ROSC admitted to the hospital.

When comparing Utstein subgroups (Table 3), the proportion of OHCAs witnessed by EMS decreased in the postpandemic period compared with the pandemic period, although survival with good neurological outcome increased in this subgroup from 9.1% to 15.7% (RR, 1.10; 95% CI, 1.04-1.17; *P* = .008). Patients belonging to the Utstein comparator group (subgroup 2A) who survived with good neurological outcome also increased from 21.6% to 27.5% (RR, 1.05; 95% CI, 1.00-1.10; *P* = .04), whereas this variable was unchanged in individuals with shockable initial rhythm and bystander CPR (subgroup 2B) (27.7% vs 24.3%; RR, 0.98; 95% CI, 0.92-1.05; *P* = .68). Survival of patients with witnessed OHCA and nonshockable initial rhythm (subgroup 2C) increased from 2.9% to 4.7% (RR, 1.07; 95% CI, 1.01-1.13; *P* = .01), with no significant difference in survival at hospital discharge with CPC category 1 or 2 in (2.3% vs 110 3.5%; RR, 1.07; 95% CI, 0.99-1.14; *P* = .05). Following the pandemic, more patients found in asystole with unwitnessed OHCA (subgroup 3) presented ROSC at hospital admission (6.1% vs 14.4%; RR, 1.12; 95% CI, 1.07-1.18; *P* < .001); however, concomitant changes were not found with regard to survival.

**Table 3.** Comparative Analysis of OHCA Care Outcomes by Subgroup

Variables	Individuals, No. (%)						Pandemic vs postpandemic		Prepandemic vs postpandemic		Prepandemic vs pandemic	
	Prepandemic		Pandemic		Postpandemic							
	No.	Missing	No.	Missing	No.	Missing	RR (95% CI)	*P* value	RR (95% CI)	*P* value	RR (95% CI)	*P* value
OHCA witnessed by EMS personnel (subgroup 1)												
No.	978	0	254	0	1090	0	NA	NA	NA	NA	NA	NA
ROSC at hospital	402 (41.1)	0	51 (20.1)	2 (0.8)	473 (43.4)	0	1.20 (1.14-1.26)	<.001	1.05 (0.96-1.13)	.29	0.43 (0.33-0.58)	<.001
Survival at hospital discharge	151 (15.4)	0	25 (9.9)	2 (0.8)	195 (17.9)	0	1.11 (1.05-1.17)	.002	1.08 (0.98-1.20)	.12	0.66 (0.45-0.97)	.03
Survival at hospital discharge with CPC 1-2	113 (11.6)	0	23 (9.1)	2 (0.8)	171 (15.7)	0	1.10 (1.04-1.17)	.008	1.17 (1.05-1.30)	.03	0.81 (0.55-1.19)	.28
Utstein comparator group (subgroup 2A)^a^												
No.	1051	2	205	0	1144	0	NA	NA	NA	NA	NA	NA
ROSC at hospital	559 (53.2)	2 (0.2)	93 (45.4)	1 (0.5)	608 (53.1)	0	1.05 (1.00-1.10)	.04	0.99 (0.92-1.10)	.95	0.76 (0.59-0.98)	.03
Survival at hospital discharge	285 (27.1)	0	49 (24.0)	1 (0.5)	349 (30.5)	0	1.05 (1.00-1.10)	.04	1.08 (0.99-1.18)	.08	0.86 (0.64-1.16)	.33
Survival at hospital discharge with CPC 1-2	212 (20.2)	0	44 (21.6)	1 (0.5)	315 (27.5)	0	1.05 (1.00-1.10)	.04	1.20 (1.10-1.31)	<.001	1.06 (0.78-1.44)	.69
Shockable initial rhythm and bystander CPR (subgroup 2B)												
No.	471	0	83	0	617	0	NA	NA	NA	NA	NA	NA
ROSC at hospital	263 (55.8)	0	44 (53.0)	0	332 (53.8)	0	1.00 (0.96-1.07)	.89	0.96 (0.87-1.08)	.51	0.90 (0.61-1.35)	.63
Survival at hospital discharge	127 (27.0)	49 (10.4)	26 (31.3)	0	169 (27.4)	29 (4.7)	0.98 (0.92-1.05)	.64	0.97 (0.87-1.09)	.64	1.04 (0.68-1.60)	.82
Survival at hospital discharge with CPC 1-2	97 (20.6)	49 (10.4)	23 (27.7)	0	150 (24.3)	29 (4.7)	0.98 (0.92-1.05)	.68	0.23 (0.19-0.27)	0.01	1.23 (0.79-1.90)	.36
Nonshockable initial rhythm and bystander witnessed OHCA (subgroup 2C)												
No.	2832	0	622	0	3128	0	NA	NA	NA	NA	NA	NA
ROSC at hospital	760	10 (0.4)	107 (17.2)	1 (0.2)	712 (22.8)	0	1.05 (1.02-1.09)	.002	0.73 (0.69-0.78)	<.001	0.43 (0.35-0.52)	<.001
Survival at hospital discharge	118 (4.2)	0	18 (2.9)	0	148 (4.7)	0	1.07 (1.01-1.13)	.01	0.91 (0.82-1.02)	.10	0.55(0.36-0.85)	<.001
Survival at hospital discharge with CPC 1-2	77 (2.7)	0	14 (2.3)	0	110 (3.5)	0	1.07 (0.99-1.14)	.05	0.95 (0.84-1.07)	.41	0.65 (0.40-1.06)	.08
Asystole and not witnessed OHCA (subgroup 3)												
No.	1093	4	250	3	1274	0	NA	NA	NA	NA	NA	NA
ROSC at hospital	163 (15.0)	4 (0.4)	15 (6.1)	3 (0.1)	184 (14.4)	0	1.12 (1.07-1.18)	<.001	0.98 (0.88-1.09)	.72	0.42 (0.26-0.69)	<.001
Survival at hospital discharge	24 (2.2)	0	1 (0.7)	0	25 (2.0)	0	1.15 (1.06-1.25)	<.001	.95 (0.72-1.25)	.70	0.21 (0.03-1.45)	.11
Survival at hospital discharge with CPC 1-2	14 (1.3)	0	1 (0.7)	0	20 (1.6)	0	1.14 (1.03-1.26)	.008	1.09 (0.82-1.45)	.53	0.36 (0.05-2.37)	.28

Abbreviations: CPC, cerebral performance category (categories 1-2 were considered good neurological outcome); EMS, emergency medical system; OHCA, out-of-hospital cardiac arrest; ROSC, return of spontaneous circulation; RR, relative risk.

^a^ Includes patients with shockable initial rhythm and bystander-witnessed OHCA.

When comparing prepandemic and postpandemic periods, we observed that some variables had not yet returned to prepandemic levels, such as ambulance arrival within both 8 (20.8% vs 16.6%; RR, 0.85; 95% CI, 0.80-0.89; *P* < .001) and 15 (59.3% vs 56.4%; RR, 0.89; 95% CI, 0.87-0.93; *P* < .001) minutes, although AED use delivering shock did (5.6% vs 6.8%; RR, 1.12; 95% CI, 1.03-1.22; *P* = .01), likely due to the use by other public services (0.9% vs 2.2%; RR, 1.79; 95% CI, 1.39-2.27; *P* < .001). A general recovery was indicated via significant improvements in most variables, with a significant increase seen from prepandemic to postpandemic in survival with CPC category 1 or 2 (7.1% vs 9.6%; RR, 1.22; 95% CI, 1.16-1.29; *P* < .001) (Table 1 and Table 2). Segmented regression outcomes also supported main findings that the number of OHCAs decreased significantly with the onset of the pandemic and recovered at its end (Figure 2).

## Discussion

This cohort study presents some of the first outcomes regarding OHCA health care in a defined country after gaining control of the COVID-19 pandemic. To our knowledge, published studies up to now have only compared outcomes pertaining to specific aspects between periods before and during the pandemic, including neurological recovery,^13^ survival following OHCAs witnessed by EMS,^19^ and enduring aspects of health care in relation to OCHA.^20^ However, even updates as important as the annual report of the American Heart Association on cardiac disease and stroke^21^ have not provided health outcomes that address recovery of the functioning of the health care system following the pandemic.

In this study, resuscitation attempts increased, whereas different links in the chain of survival improved, alongside overall survival and survival with good neurological outcome at hospital discharge. The recorded increase in the number of OHCAs with a resuscitation attempt by EMS is contradictory to that reported by other registers,^6,7,8,9^ although it is in accordance with previously reported data from Spain.^12^ This difference is likely related to the fact that a physician is present onboard mobile EMS units in Spain and to decision-making regarding the initiation of resuscitation. Such decision-making would have also had an impact on the decrease in OHCAs deemed futile for resuscitation recorded following the pandemic, moving toward levels seen in prior periods.^12,15^

The proportion of OHCAs experienced at home decreased significantly, returning to similar figures to those commonly recorded in the register used in this study.^12,15^ This aspect, together with the increase in CPR before EMS arrival, is associated with the likelihood of patient survival.^22^ Our data tentatively point to trends toward recovery in the first links of the chain of survival, with improvements emerging, in some instances, vs prepandemic outcomes.

Despite some recovery being seen in the time to EMS arrival on scene and AED use by first responders, levels had still not returned to those seen before the pandemic. Although the number of OHCAs with AED use remained low, the proportion of OHCAs receiving bystander defibrillation increased compared with the pandemic period. This may not seem to be noteworthy, when considered together with the increase in the number of OHCAs with initial shockable rhythm, but the impact on final survival is likely to be meaningful. In any case, our data show the extraordinary impact that the pandemic had on the first links in the chain of survival and the long way to go to recover and improve them.

With regard to prehospital treatment, we found that airway management via orotracheal intubation, one of the most problematic treatment actions during the pandemic, increased during the postpandemic period toward prepandemic levels. Furthermore, some recovery was seen in the resumption of donation programs for uncontrolled type IIA asystole. This is a good indicator of the recovery of the complex multilevel coordination required by such programs.

During the pandemic, hospital treatment was the least affected link, although the number of patients reached by the hospital was significantly reduced.^12^ Although statistically significant proportional changes did not emerge, due to the greater number of patients with ROSC taken to the hospital during the postpandemic period, the number of hospital treatments performed was higher, and the health care system has been able to handle a larger number of patients admitted after OHCA without any problems. Even the secondary prevention outcome of implantable cardioverter-defibrillator at hospital discharge increased significantly.

The percentage of OHCAs witnessed by EMS decreased. This finding was expected, given the increase in the total number of treated cases. Nonetheless, overall survival and survival with good neurological outcome at discharge significantly improved in this subgroup of patients. This finding stands out relative to a 2023 retrospective comparison by Kennedey et al.^19^ We observed a significant improvement in the survival of patients with nonshockable initial rhythm, although the overall number of such patients was very low. In patients with OHCA with shockable initial rhythm, similar proportions were found regarding both outcome variables. This is potentially associated with the lesser recovery of AED use and EMS response times.

Survival at hospital discharge recovered, exceeding even the proportion found before the pandemic, and, more importantly, the number of patients discharged with a good neurological outcome increased by 78%. It is well established that improvements in patient survival are dependent on all links in the chain of survival,^23^ although it must be conceded that not all of these links contribute in the same way.^24^ Improvement in OHCA survival is a slow process with periods of stagnation^24^ and lags before any impact is felt on final outcomes.^25,26^ In Eureca Two the survival results were not superior to Eureca One, which, together with the analysis of the Swedish registry, indicates the progress and setbacks shown by survival to OHCA.^27^ Furthermore, these outcomes were significantly impinged by the COVID-19 pandemic. Nonetheless, in the comparison between pandemic and prepandemic periods, general data showed the recovery capacity of the EMS, with outcomes regarding even the most demanding time-dependent process improving.

### Limitations

This study has some limitations. OHSCAR is a register of all OHCAs with a resuscitation attempt. It does not include OHCAs in which EMS did not initiate or continue resuscitation procedures, and, for this reason, real OHCA incidence cannot be estimated. Furthermore, differences in loss to follow-up of neurological outcome at discharge, especially during prepandemic period, may have partly affected findings.

## Conclusions

This cohort study found that the postpandemic era was associated with changes in OHCA care compared with prepandemic and pandemic periods. The increase in OHCAs attended to by EMS, decrease in futile resuscitation attempts, and improved care at all links of the chain of survival were associated with a meaningful increase in the number of individuals recovering from OHCA with good neurological outcome.

## Notes

Supplement 1. **eTable.** Population of the regions included in the study according to the official census of the National Institute of Statistics

Supplement 2.
OHSCAR Investigators Group members

Supplement 3.
Data Sharing Statement
