# Error in Supplement 2

**DOI:** 10.1001/jamanetworkopen.2024.2503

**Published:** 2024-02-21

In the Original Investigation titled “Out-of-Hospital Cardiac Arrest Following the COVID-19 Pandemic,”^1^ published January 23, 2024, the list of OHSCAR Investigators Group members in Supplement 2 was incomplete. This article has been corrected.^1^
