# Supplementary material for: Out-of-Hospital Cardiac Arrest Following the COVID-19 Pandemic
Source: JAMA Netw Open. 2024 Jan 23;7(1):e2352377. doi: 10.1001/jamanetworkopen.2023.52377 (PMC10807256; doi:10.1001/jamanetworkopen.2023.52377)
Supplement: Supplement 1. — eTable. Population of the regions included in the study according to the official census of the National Institute of Statistics [file jamanetwopen-e2352377-s001.pdf]

Supplemental Online Content

Ruiz Azpiazu JI, Fernández del Valle P, Sucunza AE, et al; OHSCAR investigators group. Out-of-hospital cardiac arrest following the COVID-19 pandemic. *JAMA Netw Open*. 2024;7(1):e2352377. doi:10.1001/jamanetworkopen.2023.52377

eTable. Population of the Regions Included in the Study According to the Official Census of the National Institute of Statistics

This supplemental material has been provided by the authors to give readers additional information about their work.

© 2024 Ruiz Azpiazu JI et al. *JAMA Network Open.*

eTable. Population of the regions included in the study according to the official census of the National Institute of Statistics.

Years 2018* (Pre-Covid period, PrCv), 2020*(Covid period, Cv) and 2022* (Post-Covid period, PsCv).

	**PrCv***	**Cv****	**PsCv*****
**Aragón**	1.316.040	1.330.333	1.314.159
**Castilla y León**	2.435.867	2.401.307	2.376.687
**Cataluña**	7.441.140	7.652.348	7.679.192
**Comunidad Valenciana**	3.092.512	3.143.781	3.168.235
**Pais Vasco**	2.167.283	2.189.138	2.176.918
**Extremadura**	1.077.506	1.061.979	1.053.317
**La Rioja**	312.618	315.931	315.916
**Madrid**	6.476.705	6.747.068	6.769.373
**Navarra**	640.337	656.509	659.155
**Total**	**27.670.137**	**28.200.986**	**28.204.408**

© 2024 Ruiz Azpiazu JI et al. *JAMA Network Open.*
