# Supplementary material for: Out-of-Hospital Cardiac Arrest Following the COVID-19 Pandemic
Source: JAMA Netw Open. 2024 Jan 23;7(1):e2352377. doi: 10.1001/jamanetworkopen.2023.52377 (PMC10807256; doi:10.1001/jamanetworkopen.2023.52377)
Supplement: Supplement 2. — OHSCAR Investigators Group members [file jamanetwopen-e2352377-s002.pdf]

Supplemental Online Content: Nonauthor Collaborators
*First name, last name, and suffix (if applicable) are required and will appear in PubMed.

*Group Name(s): OHSCAR investigators group											
***First Name and Middle Initial(s)**	***Last Name**	***Suffix (eg, Jr, III)**	Academic Degrees	Institution	Location (city, state/province, country)	Role or Contribution, eg, chair, principal investigator	Group (if more than 1 Group listed in the byline) and/or Subgroup (eg, Steering Committee)				
Luis	Olavarria Govantes		MD	Centro de Emergencias 061, SP M	Andalucía, España	Co-investigator. Data acquisition and quality control in the corresponding EMS.					
María J	Luque-Hernández		MD	Centro de Emergencias 061, SP M	Andalucía, España	Co-investigator. Data acquisition and quality control in the corresponding EMS.					
Miguel A	Paz-Rodríguez		MD	Centro de Emergencias 061, SP Hu	Andalucía, España	Co-investigator. Data acquisition and quality control in the corresponding EMS.					
Fernando	Ayuso-Batista		MD	Centro de Emergencias 061, SP Có	Andalucía, España	Co-investigator. Data acquisition and quality control in the corresponding EMS.					
Carmen	Gutiérrez-García		MD	Centro de Emergencias 061, SP M	Andalucía, España	Co-investigator. Data acquisition and quality control in the corresponding EMS.					
María R	Soto-García		MD	Centro de Emergencias 061, SP M	Andalucía, España	Co-investigator. Data acquisition and quality control in the corresponding EMS.					
María A	Caballero-García		MD	Centro de Emergencias 061, SP Se	Andalucía, España	Co-investigator. Data acquisition and quality control in the corresponding EMS.					
Roberto	Antón Ramas		MD	061 e Instituto de Ciencias de la Salud	Aragón, España	Co-investigator. Data acquisition and quality control in the corresponding EMS.					
Marcel	Chueca García		MD	061 e Instituto de Ciencias de la Salud	Aragón, España	Co-investigator. Data acquisition and quality control in the corresponding EMS.					
Ignacio	González Herráiz		MD	061 e Instituto de Ciencias de la Salud	Aragón, España	Co-investigator. Data acquisition and quality control in the corresponding EMS.					
Maria I	Ceniceros-Rozalen		MD	SAMU061	Islas Baleares, España	Co-investigator. Data acquisition and quality control in the corresponding EMS.					
Esther	Arias Moya		MD	SAMU061	Islas Baleares, España	Co-investigator. Data acquisition and quality control in the corresponding EMS.					
María N	Gonzalez Quintana		MD	Servicio de urgencias Canario	Islas Canarias, España	Co-investigator. Data acquisition and quality control in the corresponding EMS.					
Faustino	Redondo Revilla		MD	Servicio de urgencias Canario	Islas Canarias, España	Co-investigator. Data acquisition and quality control in the corresponding EMS.					
