# Supplementary material for: Out-of-Hospital Cardiac Arrest Following the COVID-19 Pandemic
Source: JAMA Netw Open. 2024 Jan 23;7(1):e2352377. doi: 10.1001/jamanetworkopen.2023.52377 (PMC10807256; doi:10.1001/jamanetworkopen.2023.52377)
Supplement: Supplement 3. — Data Sharing Statement [file jamanetwopen-e2352377-s003.pdf]

Data Sharing Statement

Ruiz Azpiazu. Out-of-Hospital Cardiac Arrest Following the COVID-19 Pandemic. *JAMA Netw Open*. Published January 23, 2024. doi:10.1001/jamanetworkopen.2023.52377

Data
Data available: Yes
Data types: Deidentified participant data, Data dictionary
How to access data: Data used for the present article can be shared with patients as alphanumeric tables, following written requests outlining the aim and treatment of requested data. Such requests will be addressed to: Dr. Rosell Ortiz (frosell@riojasalud.es) and Dr. Ruiz Azpiazu (Jiruiz@riojasalud.es). As a formal requirement, all requests must meet Spanish legislation regarding handling healthcare data. In all cases, once this formality is satisfied, there will be no issue in arranging a consultation and external review.
When available: With publication

Supporting Documents
Document types: Statistical/analytic code
How to access documents: All data requested must be addressed to Dr. Rosell Ortiz (frosell@riojasalud.es) and Dr. Ruiz Azpiazu (Jiruiz@riojasalud.es)
When available: With publication

Additional Information
Who can access the data: researchers whose proposed use of the data has been approved.
Types of analyses: for a pre-specify purpose
Mechanisms of data availability: after approval of a proposal with a signed data access agreement
